# The change in right ventricular systolic function according to the revascularisation method used, following acute ST -segment elevation myocardial infarction

**DOI:** 10.5860/CVJA-2015-077

**Published:** 2016

**Authors:** Ilker Gul, Mustafa Zungur, Aysel Islamli, Ahmet Cagri Aykan, Ezgi Kalaycioğlu, Turhan Turan, Tayyar Gokdeniz, Mustafa Beyazit Alkan, Ahmet Sayin, Murat Bilgin

**Affiliations:** Department of Cardiology, Faculty of Medicine, Şifa University, Izmir, Turkey; Department of Cardiology, Faculty of Medicine, Şifa University, Izmir, Turkey; Department of Cardiology, Faculty of Medicine, Şifa University, Izmir, Turkey; Department of Cardiology, Ahi Evren Thoracic and Cardiovascular Surgery Training and Research Hospital, Trabzon, Turkey; Department of Cardiology, Ahi Evren Thoracic and Cardiovascular Surgery Training and Research Hospital, Trabzon, Turkey; Department of Cardiology, Ahi Evren Thoracic and Cardiovascular Surgery Training and Research Hospital, Trabzon, Turkey; Department of Cardiology, Faculty of Medicine, Kafkas University, Kars, Turkey; Department of Cardiology, Acıpayam State Hospital, Denizli, Turkey; Department of Cardiology, Tepecik Education and Research Hospital, Izmir, Turkey; Department of Cardiology, Dışkapı Yıldırım Beyazıt Training and Research Hospital, Ankara, Turkey

**Keywords:** ST-elevation myocardial infarction (STEMI), primary percutaneous coronary intervention (PPCI), thrombolytic therapy (TT), right ventricular systolic function

## Abstract

**Objective:**

The level of right ventricular (RV) systolic function has prognostic importance in right ventricular ST-segment elevation myocardial infarction (RV-STEMI). This study aimed to evaluate the changes in RV systolic function in patients with RV-STEMI according to the revascularisation method used for their management.

**Methods:**

The first group consisted of 132 patients who received primary percutaneous coronary intervention (PPCI). The 78 patients who had received thrombolytic therapy (TT) in external centres before referral to our centre for PCI within three to 12 hours of RV-STEMI were included in the second group. All patients were evaluated by conventional and two-dimensional speckle-tracking echocardiography.

**Results:**

There were 172 male patients and their mean age was 63.7 ± 11.8 years. There were no significant differences between the two groups with regard to right ventricular systolic parameters at admission and at the one-month follow-up visit. The echocardiographic changes between admission and the one-month follow up were investigated for the patients included in the study groups. Mean values of each parameter observed at the one-month follow up were significantly increased compared to those at admission within each group.

**Conclusion:**

Our study demonstrated that PCI within three to 12 hours following TT provided similar benefits on right ventricular systolic function compared to PPCI in patients with RV-STEMI.

## Objective

ST-elevation myocardial infarction (STEMI) is characterised by a loss of contractile tissue and a change in ventricle geometry that causes substantial impairment of the ventricular systolic and diastolic functions.^1^ In coronary artery disease (CAD), left ventricular (LV) function has been widely evaluated by means of echocardiographic methods. LV function has long been known to be among the most important determinants of morbidity and mortality.^2^,^3^ However, the right ventricle (RV) has been the subject of fewer studies compared to the left ventricle.

RV-STEMI has been reported in 10 to 60% of patients with inferior STEMI.^4^-^6^ The co-existence of inferior STEMI and RV-STEMI has been shown to increase morbidity and mortality rates.^7^,^8^

The time lapse between the onset of symptoms and admission to hospital is known as the symptom-to-door time. Myocardial damage and cardiac complications are more likely to progress with prolonged periods without intervention after STEMI.^9^,^10^ Current guidelines emphasise the benefits of reperfusion within the first 12 hours after STEMI.

Successful reperfusion within the first three hours is reported to provide improved prognosis. For this purpose, the selection of appropriate reperfusion strategy is an important discussion topic. Reperfusion strategies include fibrinolysis and primary percutaneous coronary intervention (PPCI) techniques. PPCI is preferable in a 24-hours-a-day, seven-days-a-week centre with an established coronary intervention facility.

When the transfer time from centres without coronary intervention laboratories does not exceed 120 minutes and the door-to-balloon time does not exceed 90 minutes, PPCI is again the preferred method for revascularisation. If these requirements are not met, fibrinolytic therapy may be performed for the patients.

RV-STEMI requires immediate revascularisation in affected patients. Revascularisation can be achieved with methods such as percutaneous coronary intervention or thrombolytic therapy. Current guidelines recommend the appropriate treatment of coronary arteries after performing coronary angiography within three to 24 hours following TT. 11,12

This study aimed to evaluate the effects of coronary intervention on right ventricular systolic function by comparing PPCI with PCI performed within three to 12 hours of TT, as assessed by transthoracic echocardiography, in patients with RV-STEMI.

## Methods

This prospective, observational cohort study was carried out between January 2012 and February 2013. We interviewed 210 patients who met the criteria for admission and had had inferior myocardial infarction with right ventricular involvement (RV-STEMI) for the first time. RV-STEMI was defined as new ST-segment elevation ≥ 0.1 mV at the J-point in two contiguous inferior leads accompanied by new ST-segment elevation ≥ 0.1 mV in the right ventricular leads (V3R–V4R).

Patients who had infection, heart muscle disease or chronic inflammatory disease were not included in the study. At hospital admission, the patients who had cardiogenic shock, chronic pulmonary disease or systolic pulmonary artery pressure > 35 mmHg, renal failure (creatinine > 2.5 mg/dl) or a history of cerebrovascular events were also excluded from the study. Since RV systolic function was evaluated in our study, those who had diseases such as pulmonary hypertension that could impair the RV systolic function were excluded from the study. As right heart catheterisation could not be applied during primary PCI, the patients with echocardiographically measured systolic pulmonary arterial pressure > 35 mmHg were not included in the study. Patients with unknown time of symptom onset or a StD time longer than 12 hours were also not included in the study.

The patients were divided into two groups according to admission to hospital; 132 patients who underwent PPCI were identified as the first group. Seventy-eight patients who underwent PCI in our centre within three to 12 hours after receiving TT in other centres were included in the second group.

Our centre is a tertiary hospital and coronary intervention facilities are available 24 hours a day, seven days a week. Patient records are kept on a regular basis, starting at admission to the emergency department. In addition to these data, the onset of symptoms was sought from the patients themselves or their relatives.

The exact time of patient admission in the emergency department was identified as door-time. Symptom-to-door (StD) time was determined by calculating the difference between the two periods. The exact time the patient’s coronary balloon had been inflated was recorded in the angiography laboratory. The time lapse from the patient’s admission to the emergency department to inflating the balloon was calculated as doorto- balloon time (DtB). Symptom-to-balloon (StB) time was calculated in addition to StD and DtB. Symptom-to-needle (StN) time was calculated as the time from the initial onset of symptoms to the start of thrombolytic drug administration. The time lapse from the patient’s admission to the emergency department to the start of thrombolytic drug administration was calculated as door-to-needle time (DtN).

During the study, intervention was performed only for the culprit arteries responsible for the infarction. Elective interventions were performed for the other lesions. The invasive treatment methods applied during PCI, and balloon and stent diameters and lengths were recorded. The diameters of the vessels with lesions were calculated from the coronary angiography examinations.

Traditional variables that have been used to assess response to TT were decrease in chest pain, ST-segment resolution and reperfusion arrhythmias. Patients who had < 50% ST-segment resulution were excluded from the study. Patients who required rescue PCI, patients scheduled for coronary artery bypass grafting (CABG) or those who underwent percutaneous intervention for all critical lesions due to haemodynamic instability were also excluded from the study. Patients with a DtB time longer than 30 minutes in the PPCI group, and those with a door-to-needle (DtN) time longer than 30 minutes in the TT group were not included in the study.

All patients received dual antiplatelet therapy with acetylsalicylic acid and clopidogrel (300–600 mg) loading dose before coronary intervention. Peri-procedural anticoagulation consisted of intravenous unfractioned heparin (70 IU/kg) in all cases. Clopidogrel (75 mg per day) and acetylsalicylic acid (100 mg per day) were prescribed for at least one year. Blood samples were collected from each subject immediately after presenting at the emergency department. Cardiac enzymes, liver function tests, kidney function tests, complete blood count and thyroid function tests were performed on these samples.

## Echocardiography

A Vivid-S5 echocardiography device is readily available in the emergency department of our centre (General Electric Vingmed Ultrasound, Horten, Norway, with a 3.6-MHz transducer). Echocardiographic evaluation is performed rapidly in all patients presenting at the emergency room (ER) with acute coronary syndrome (ACS). Imaging is performed by the echocardiography operator simultaneously while patients with STEMI are prepared for coronary intervention. In our study, prolonged StD durations were avoided in order to assess echocardiographic parameters.

Echocardiographic evaluation of the RV is more difficult than that of the LV. An appropriate imaging window may not be achieved due to restrictions caused by the sternum and other anatomical structures. Therefore, patients with inadequate echocardiographic imaging quality were excluded from the present study.

American Society of Echocardiography (ASE) recommendations were followed for the evaluation of RV systolic function. RV end-systolic and end-diastolic diameters were measured from the left parasternal long-axis view. RV basal, mid and longitudinal diameters were measured from the apical four-chamber view. Endocardium margins were drawn from the tricuspid annulus to the RV apex and from there to the opposite side of the tricuspid annulus in the apical four-chamber view in order to calculate RV fractional area change (RV-FAC). End-systole and end-diastole areas were individually calculated with this approach. RV-FAC was determined by means of the formula:

RV-FAC = end-diastole area – end-systole area/end-diastole area

Tricuspid annular plane systolic excursion (TAPSE) was calculated by measuring the movement magnitude of the RV annular segment in the longitudinal plane using the M-mode method. This measurement was performed on the apical fourimaging window of the tricuspid lateral annulus.

Right ventricle isovolumic acceleration (RV-IVA) was calculated by dividing the peak isovolumic myocardial velocity calculated at the time of isovolumic contraction by the time to peak velocity using tissue Doppler on the lateral tricuspid annulus. RV-S′ was calculated by measuring systolic velocity using tissue Doppler on the right ventricular lateral tricuspid annulus.

Right ventricle myocardial performance index (RV-MPI) is one of the methods recommended for the evaluation of global RV function. It was calculated by dividing the sum of isovolumic contraction time (IVCT) and isovolumic relaxation time (IVRT) by the tricuspid ejection time: 13-17

RV-MPI = IVRT + IVCT/ET

Left ventricular ejection fraction (LVEF) was calculated from the four- and two-chamber views using the modified Simpson biplane method. LV wall-motion score index (LV-WMSI) was calculated according to the 16-segment model of the American Society of Echocardiography. In accordance with this model, normokinesis, mild-moderate hypokinesis, severe hypokinesis, akinesia and dyskinesia were evaluated with the scores 1, 2, 3, 4 and 5, respectively. The total value was divided into the evaluated segment number and WMSI was obtained.^18^

Echocardiographic examinations were performed by the same investigators, who were blinded to the patients’ data, at baseline and after the first month. All measurements were calculated from three consecutive cycles, and the average of the three measurements was recorded.

## Speckle-tracking echocardiography

Two-dimensional speckle-tracking echocardiography (2D STE) is a novel technique used for the measurement of cardiac mechanics. It assesses myocardial deformation and the myocardial deformation rate by tracking speckles in the myocardium on grayscale (B-mode) images, and can be used to evaluate both global and regional myocardial strain and strain rate.^19^,^20^

The investigations were performed with the patients in the left lateral decubitus position, in the parasternal and apical four-chamber views. Digital routine grayscale 2D ciné loops and tissue Doppler ciné loops were obtained from three consecutive beats with end-expiratory apnoea from standard apical fourand two-chamber views. Frame rates of 70–90 Hz were used for routine grayscale imaging in the speckle-tracking analysis. Sector width was optimised to allow for complete myocardial visualisation while maximising the frame rate. Gain settings were adjusted for routine clinical grayscale 2D imaging to optimise endocardial definition.

Longitudinal deformation in the RV free wall was assessed by 2D speckle-tracking longitudinal strain using a routine grayscale RV focused-view image, which was performed offline with dedicated software (EchoPAC 108.1.12, General Electric- Vingmed Medical Systems, Horten, Norway) by one experienced cardiologist blinded to data about the patients’ status.

Briefly, a region of interest (ROI) was traced with a point-and-click approach on the endocardium at end-diastole in the RV from the RV focused view. A second, larger ROI was then generated and manually adjusted near the epicardium. The RV was divided into six standard segments (at the basal, middle and apical levels), and six corresponding time–strain curves were generated. RV free-wall longitudinal speckle-tracking strain (RV-free-S) was calculated by averaging each of the three regional peak systolic strains along the entire RV free wall and RV free systolic strain rate (RV-free-SR), were calculated in the same manner.

The patients were prospectively followed during the in-hospital period and first month after RV-STEMI. Informed consent was obtained from each subject, and the study was conducted in accordance with the Helsinki Declaration. The study protocol was approved by the ethics committee.

## Variability analysis

Intra- and inter-observer variability were assessed in the echocardiographic data obtained from a subgroup of 30 subjects. One month later, the first operator repeated the analysis to assess intra-observer variability. To assess inter-observer variability, the second operator who was unaware of the previous measurements, analysed the rotational parameters two days later.

Agreement analysis for inter- and intra-observer variability of RV measurements revealed a high level of agreement, with a mean difference of 0.18 (95% limit of agreement –0.5, 0.96). For intra-observer variabilities, intraclass correlation coefficient of RV-free-ST and RV-free-STR-S were 0.907 (95% CI 0.840–0.943) and 0.954 (95% CI 0.823–0.967), respectively. 

## Statistical analysis

SPSS 17 (SPSS Inc, Chicago, IL, USA) was used for statistical analysis. The Kolmogorov–Smirnov test was used to evaluate whether the numerical variables were normally distributed. For data showing an abnormal distribution, median and interquartile ranges were displayed. Continuous variables were presented as mean ± standard deviation, and categorical ones were presented as percentage (%).

The two study groups were compared using the Student’s *t*-test or Mann–Whitney U- and chi-squared or Fisher’s exact tests, as appropriate. In each group, follow-up comparisons (early period and one month) were performed using the paired *t*-test and Wilcoxon rank test, as appropriate. Intraclass correlation coefficients and Bland–Altman analysis were used for echocardiographic measurements to assess intra- and interobserver reproducibility, respectively. A *p*-value < 0.05 was considered statistically significant.

## Results

There were 172 male patients in the study and the mean age was 63.7 ± 11.8 years. One-month clinical follow up was available in all 172 patients (100.0%). There was no difference between the groups in terms of age, diabetes, hypertension and smoking status, and length of hospital stay. Average values were similar for heart rate, and systolic and diastolic blood pressure. Creatinine and troponin I values were higher in the TT group. No difference was found between the groups with regard to blood glucose and haemoglobin levels, and platelet count.

The StD time from the onset of symptoms to admission to hospital was similar across the patients who had received similar medical treatment. Average DtB duration was 20.2 minutes for the PPCI group. DtN time was 23.2 minutes and StN time was 291.7 minutes in the TT group.

Although the mortality rate in the first month was higher in the TT group, the difference was not statistically significant (p = 0.077). Re-MI and re-hospitalisation rates were similar between the groups during the first month. There were more ventricular tachycardia (p = 0.039) and ventricular fibrillation (p = 0.005) cases in the TT group. Rate of atrioventricular block of at least second degree, observed during the hospital stay, was similar. Rates of TIMI 0 and TIMI I–II flow following PCI were similar across the groups. The groups were similar in terms of SYNTAX score, one of the indicators of extent and complexity of coronary lesion (Table 1).

**Table 1:** General characteristics, cardiac complications and laboratory results of the patients according to the study groups

*Parameter*	*PPCI (n = 132)*	*TT + PCI (n = 78)*	*p-value*
Age (years)	64.3 (± 12.1)	62.9 (±10.2)	0.128
Diabetes mellitus, n (%)	25 (18.9)	21 (26.9)	0.120
Hypertension, n (%)	73 (55.3)	41 (52.6)	0.404
Current smoking, n (%)	64 (48.5)	47 (60.3)	0.066
Heart rate (beats/min)	61.8 (± 21.1)	63.9 (± 20.72)	0.480
SBP (mmHg)	123.9 (± 29.5)	128.6 (± 34.12)	0.292
DBP (mmHg)	75.1 (± 17.3)	78.4 (± 20.2)	0.206
Creatinine (mg/dl)	0.98 (± 0.39)	1.16 (± 0.83)	0.048
Peak troponin I (ng/dl)	61.7 (41.2–105.3)	88.3 (48.3–166.8)	0.006
Blood glucose (mg/dl)	151.4 (87.5–245.3)	164.5 (92.7–261.3)	0.251
Haemoglobin (mg/dl)	13.5 (± 1.9)	13.6 (± 1.7)	0.521
Platelets (103/μl)	245.9 (± 59.9)	253.1 (± 53.3)	0.465
Clopidogrel, n (%)	132 (100)	78 (100)	1.000
Acetylsalicylic acid, n (%)	132 (100)	78 (100)	1.000
Beta-blocker, n (%)	108 (81.8)	60 (76.9)	0.247
Statin, n (%)	131 (99.2)	77 (98.7)	0.983
ACE/ARB, n (%)	118 (89.4)	66 (84.6)	0.131
Total mortality, n (%)	8 (6.1)	7(8.9)	0.557
VT, n (%)	18 (13.6)	19 (24.4)	0.039
VF, n (%)	3 (2.2)	9 (11.5)	0.005
High-degree AV block, n (%)	16 (12.1)	8 (10.2)	0.246
Post-PCI TIMI 0 flow rate, n (%)	3 (2.3)	1 (1.3)	0.618
Post-PCI TIMI I–II flow rate, n (%)	9 (6.8)	4 (5.1)	0.643
Pre-PCI SYNTAX score	22.1 (± 8.8)	21.2 (± 21.1)	0.511
Post-PCI SYNTAX score	8.4 (± 8.6)	9.1 (± 7.2)	0.568
Intensive care unit (days)	3.58 ± 1.19	3.85 ± 2.21	0.252
Duration of hospital stay (days)	6.48 ± 3.17	7.03 ± 4.71	0.322
Symptom-to-door time (min)	285.9 ± 135.1	268.5 ± 141.8	0.577
Mortality in hospital, n (%)	6 (4.6)	5 (6.4)	0.108
Mortality rate after discharge from hospital, n (%)	2 (1.5)	2 (2.5)	0.591
Re-MI after discharge from hospital, n (%)	2 (1.5)	2 (2.5)	0.591
Re-hospitalisation, n (%)	4 (3.0)	3 (3.8)	0.856

ACE: angiotensin converting enzyme; ARB: angiotensin receptor blocker; AV: atrioventricular; DBP: diastolic blood pressure; MI: myocardial infarction; PCI: percutaneous coronary intervention; PPCI: primary percutaneous coronary intervention; SBP: systolic blood pressure; DBP: diastolic blood pressure; TT: thrombolytic therapy; VT: ventricular tachycardia; VF: ventricular fibrillation.

There was no difference between the two groups regarding RV dimensions, volumes and function on admission to our hospital. There was no difference between the two groups with regard to the conventional RV systolic parameters, RV dimensions and volumes at the one-month follow up. There was no difference between the groups regarding the LVEF and LV-WMSI on admission to hospital and at the one-month follow up (Table 2).

**Table 2:** Right and left ventricular echocardiographic parameters according to the groups

	*First 12 hours (early period)*			*First month after RV-STEMI*		
*Parameter*	*PPCI*	*TT+PCI*	*p-value*	*PPCI*	*TT+PCI*	*p-value*
RV basal (mm)	37.3 (± 4.4)	37.9 (± 5.1)	0.325	34.3 (± 6.1)	33.8 (± 7.4)	0.128
RV mid (mm)	31.8 (± 2.4)	31.2 (± 3.5)	0.240	26.9 (± 3.6)	27.5 (± 2.8)	0.838
RV longitudinal (mm)	72.2 (± 5.4)	73.1 (± 2.4)	0.486	65.3 (± 2.4)	66.1 (± 2.4)	0.214
RV-eDV indexed (ml/m^2^)	64.3 (± 9.4)	63.2 (± 5.6)	0.082	56.9 (± 4.7)	57.1 (± 8.7)	0.762
RV-eSV indexed (ml/m^2^)	37.1 (± 4.3)	38.6 (± 3.9)	0.844	30.1 (± 2.9)	31.9 (± 3.0)	0.274
RVEF (%)	43.1 (± 8.3)	42.6 (± 8.8)	0.978	49.9 (± 7.2)	49.3 (± 7.2)	0.934
RV-FAC (%)	29.9 (± 7.4)	29.4 (± 7.4)	0.458	33.3 (± 6.8)	34.2 (± 6.8)	0.110
RV-TAPSE (mm)	16.1 (± 4.0)	16.7 (± 4.2)	0.082	22.4 (± 3.8)	21.8 (± 3.9)	0.102
RV-MPI	0.49 (± 0.09)	0.50 (± 0.12)	0.648	0.41 (± 0.12)	0.40 (± 0.14)	0.365
RV-IVA (m/s^2^)	2.24 (± 0.64)	2.31 (± 0.52)	0.121	2.99 (± 0.63)	2.90 (± 0.55)	0.071
RV-S′ (cm/s)	9.1 (± 1.7)	9.5 (± 1.1)	0.212	11.3 (± 3.2)	10.9 (± 3.2)	0.094
RV apical strain (%)	–9.7 (± 1.8)	–10.1 (± 1.5)	0.286	–16.6 (± 2.9)	–15.9 (± 2.1)	0.293
RV mid strain (%)	–14.1 (± 2.3)	–14.9 (± 1.9)	0.351	–22.6 (± 3.6)	–21.2 (± 2.1)	0.102
RV basal strain (%)	–18.2 (± 4.7)	–17.6 (± 2.0)	0.422	–24.8 (± 4.1)	–24.2 (± 2.1)	0.434
RV free strain (%)	–14.0 (± 2.7)	–14.2 (± 1.7)	0.429	–21.3 (± 3.3)	–20.4 (± 3.7)	0.102
RV apical strain rate (1/s)	–0.8 (± 0.4)	–0.9 (± 0.3)	0.424	–1.5 (± 0.5)	–1.4 (± 2.1)	0.624
RV mid strain rate (1/s)	–1.1 (± 0.6)	–1.0 (± 0.5)	0.435	–2.2 (± 0.4)	–2.1 (± 2.1)	0.743
RV basal strain rate (1/s)	–1.7 (± 0.6)	–1.6 (± 0.4)	0.515	–2.3 (± 0.5)	–2.2 (± 2.1)	0.802
RV free strain rate (1/s)	–1.2 (± 0.4)	–1.2 (± 0.5)	0.512	–2.0 (± 0.4)	–1.9 (± 0.6)	0.292
LVEF (%)	49.3 (± 8.3)	48.4 (± 7.6)	0.094	52.2 (± 6.6)	51.9 (± 8.3)	0.498
LV-WMSI	1.48 (± 0.27)	1.52 (± 0.34)	0.413	1.16 (± 0.25)	1.21 (± 0.31)	0.128

eDV: end-diastolic volume; EF: ejection fraction; eSV: end-systolic volume; FAC: fractional area change; IVA: isovolumic acceleration; LV: left ventricle; MPI: myocardial performance index; RV: right ventricle; S′: tissue Doppler systolic wave; TAPSE: tricuspid annulus planimetric systolic excursion; WMSI: wall motion score index.

Echocardiographic changes between admission and one-month follow up were investigated for the patients included in the study groups. Mean values of each parameter were significantly increased at the one-month follow up compared to the early period within each individual group (Table 3).

**Table 3:** Right ventricular systolic parameters according to the echocardiographic evaluation periods

	*PPCI*			*TT+PCI*		
*Parameter*	*Early period*	*First month*	*p-value*	*Early period*	*First month*	*p-value*
RV basal (mm)	37.3 (± 4.4)	34.3 (± 6.1)	0.042	37.9 (± 5.1)	33.8 (± 7.4)	0.018
RV mid (mm)	31.8 (± 2.4)	26.9 (± 3.6)	0.005	31.2 (± 3.5)	27.5 (± 2.8)	0.026
RV longitudinal (mm)	72.2 (± 5.4)	65.3 (± 2.4)	0.038	73.1 (± 2.4)	66.1 (± 2.4)	0.032
cRV-eDV indexed (ml/m^2^)	64.3 (± 9.4)	56.9 (± 4.7)	0.006	63.2 (± 5.6)	57.1 (± 8.7)	0.012
RV-eSV indexed (ml/m^2^)	37.1 (± 4.3)	30.1 (± 2.9)	0.008	38.6 (± 3.9)	31.9 (± 3.0)	0.017
RVEF (%)	43.1 (± 8.3)	49.9 (±7.2)	< 0.001	42.6 (± 8.8)	49.3 (± 7.2)	< 0.001
RV-FAC (%)	29.9 (± 7.4)	33.3 (± 6.8)	0.011	29.4 (± 7.4)	34.2 (± 6.8)	0.006
RV-TAPSE (mm)	16.1 (± 4.0)	22.4 (± 3.8)	< 0.001	16.7 (± 4.2)	21.8 (± 3.9)	< 0.001
RV-MPI	0.49 (± 0.09)	0.41 (± 0.12)	< 0.001	0.50 (± 0.12)	0.40 (± 0.14)	< 0.001
RV-IVA (m/s^2^)	2.24 (± 0.64)	2.99 (± 0.63)	< 0.001	2.31 (± 0.52)	2.90 (± 0.55)	< 0.001
RV-S′ (cm/s)	9.1 (± 2.2)	11.3 (± 3.0)	< 0.001	9.5 (± 1.1)	10.9 (± 3.0)	0.003
RV apical strain (%)	–9.7 (± 1.8)	–16.6 (± 2.9)	< 0.001	–10.1 (± 1.5)	–15.9 (± 2.1)	< 0.001
RV mid strain (%)	–14.1 (± 2.3)	–22.6 (± 3.6)	< 0.001	–14.9 (± 1.9)	–21.2 (± 2.1)	< 0.001
RV basal strain (%)	–18.2 (± 4.7)	–24.8 (± 4.1)	0.006	–17.6 (± 2.0)	–24.2 (± 2.1)	0.003
RV free strain (%)	–14.0 (± 2.7)	–21.6 (± 3.3)	< 0.001	–14.2 (± 1.7)	–21.1 (± 3.7)	< 0.001
RV apical strain rate (1/s)	–0.8 (± 0.4)	–1.5 (± 0.5)	< 0.001	–0.9 (± 0.3)	–1.4 (± 2.1)	0.004
RV mid strain rate (1/s)	–1.1 (± 0.6)	–2.2 (± 0.4)	< 0.001	–1.0 (± 0.5)	–2.1 (± 2.1)	< 0.001
RV basal strain rate (1/s)	–1.7 (± 0.6)	–2.3 (± 0.5)	0.002	–1.6 (± 0.4)	–2.2 (± 2.1)	< 0.001
RV free strain rate (1/s)	–1.22 (± 0.4)	–2.11 (± 0.4)	< 0.001	–1.29 (± 0.5)	–1.96 (± 0.6)	< 0.001
LVEF (%)	49.3 (± 8.3)	52.2 (± 6.6)	0.048	48.4 (± 7.6)	51.9 (± 8.3)	0.040
LV-WMSI	1.48 (± 0.27)	1.16 (± 0.25)	< 0.001	1.52 (± 0.34)	1.21 (± 0.31)	< 0.001

eDV: end diastolic volume; EF: ejection fraction ; eSV: end-systolic volume; FAC: fractional area change; IVA: isovolumic acceleration; LV: left ventricle; MPI: myocardial performance index; RV: right ventricle; S′: tissue Doppler systolic wave; TAPSE: tricuspid annulus planimetric systolic excursion; WMSI: wall motion score index.

2D-STE analysis of the RV revealed that the study groups had similar RV-free-S and RV-free-SR levels in the early period and at the one-month follow up (Tables 2, 3). According to these results, the strain/strain rates of the RV segments in both groups were significantly lower in the early period than those at the one-month follow up. In addition, changes in the RV mean strain/strain rates were significantly different (Table 3).

Evaluation of the angiographic parameters in the PPCI and TT groups revealed a higher number of patients with singlevessel disease in the TT group. Triple-vessel disease was more common in the PPCI group. The groups were similar in terms of bifurcation lesion, left dominance, drug-eluting stent use, direct stenting, coronary ectasia, thrombus aspiration and tirofiban infusion. Similar balloons were used with regard to diameters and lengths. Increased stent diameters (*p* = 0.015) and lengths (0.005) were observed in the PPCI group (Table 4).

**Table 4:** Angiographic findings and coronary intervention characteristics between the groups

**	*PPCI (n = 132)*	*TT+PCI (n = 78)*	*p-value*
One-vessel coronary disease, n (%)	32 (24.2)	29 (37.2)	0.045
Two-vessel coronary disease, n (%)	52 (39.4)	31 (39.8)	0.820
Three-vessel coronary disease, n (%)	48 (36.4)	18 (23.1)	0.031
LMCA stenosis, n (%)	10 (7.5)	5 (6.4)	0.675
RCA proximal occlusion, n (%)	42 (31.8)	28 (35.9)	0.162
Thrombus aspiration, n (%)	13 (9.8)	6 (8.1)	0.443
Bifurcation lesion, n (%)	19 (14.4)	17 (23.0)	0.087
Left dominance, n (%)	9 (6.8)	8 (10.8)	0.285
Drug-eluting stents, n (%)	22 (16.7)	14 (19.2)	0.236
Direct stent implantation, n (%)	24 (18.2)	14 (19.2)	0.500
Coronary ectasia, n (%)	6 (4.5)	5 (6.4)	0.387
Balloon diameter (mm)	2.46 ± 0.38	2.54 ± 0.49	0.681
Balloon length (mm)	18.1 ± 4.03	17.6 ± 3.56	0.130
Stent diameter (mm)	3.18 (2.50–4.25)	2.96 (2.55–4.05)	0.015
Stent length (mm)	28.6 (14.5–38.5)	23.0 (15.3–34.8)	0.005

LMCA: left main coronary artery; PCI: percutaneous coronary intervention; PPCI: primary percutaneous coronary intervention; TT: thrombolytic therapy.

## Discussion

The results of this study demonstrated similar levels of improvement in RV function among patients managed with PCI within three to 12 hours from TT, and those managed with PPCI following RV-STEMI.

There are several conventional methods of assessing RV systolic function that should be incorporated into a routine echocardiographic assessment. These are FAC, TAPSE, RV-S′, and MPI. It is strongly recommended that at least one of the above quantitative measures be incorporated into the routine echocardiographic examination. 2D-derived estimation of RV ejection fraction is not recommended because of the heterogeneity of methods and the numerous anatomical assumptions.^13^

RV-FAC is one of the parameters recommended for the assessment of systolic function. However, this technique is dependent on imaging and the operator’s skill. Normal values of RV-FAC are accepted as > 35%.^13^ RV-FAC has been shown to correlate with RVEF in studies performed using magnetic resonance imaging (MRI). Heart failure, sudden death, stroke and pulmonary embolism have also been shown to predict mortality.^13^,^19^

In the present study, there was no difference between the groups with regard to mean RV-FAC values obtained before PCI and at the one-month follow up. Mean RV-FAC values observed at the one-month follow up were significantly increased within each group compared to the pre-PCI period.

Isovolumic acceleration (IVA) is considered a useful method to evaluate RV systolic function.^20^ However this method is not without disadvantages. It is angle-dependent and may be influenced by age and heart rate. The lower limit of pulse wave with tissue Doppler was accepted as 2.2 m/s^2^, as per the guideline recommendations.^13^

In our study, there was no difference between the groups in terms of pre-PCI RV-IVA. Although pre-PCI, RV-IVA levels were low in the two groups, mean levels were improved to normal at the one-month follow up in both groups. The difference between the early period and the one-month follow up was significant in each group.

MPI may be used for the assessment of global heart function.^21^ It enables evaluation of both systolic and diastolic function. Reduced ventricular systolic function shortens the ejection time, leading to increased MPI. MPI > 0.4 with pulse Doppler and MPI > 0.55 with tissue Doppler are considered direct indicators of impaired RV function.^13^ A normal MPI value is 0.28 ± 0.04 for the RV, and 039 ± 0.05 for the LV.^22^

In a study by Karakurt *et al.*, patients who were managed with PPCI following non-anterior STEMI were compared to those who received TT alone, and similar mean RV-MPIs were observed in both groups at 72 hours after the infarction.^23^ In our study, there was no difference between the groups in terms of mean RV-MPI values observed before percutaneous intervention and at the one-month follow up. However, the mean RV-MPI values were significantly improved during the later period compared to the early period.

TAPSE is easily obtainable and is a measure of RV longitudinal function.^13^ The preferred method to evaluate RV systolic function is often TAPSE, which is known to correlate with RVEF.^24^ TAPSE > 15 mm is reported to substantially decrease mortality rates.^14^,^25^ As Hayrapetyan *et al.* have shown, TAPSE < 14 during the 24 hours following RV-STEMI is associated with a poor prognosis.^26^ In our study, mean TAPSE values were improved at the one-month follow up in both groups. Mean TAPSE was similar between the groups in the early period and at the one-month follow up.

RV-S′ is a very reliable and easily measured parameter in young adults. However, it may fail to fully reflect systolic function in the elderly. It can be measured from the tricuspid lateral annulus by means of tissue Doppler. RV-S′ < 10 cm is associated with RV systolic dysfunction.^13^,^23^,^27^ In our study there were no significant differences between the groups in the early period and at the one-month follow up according to mean RV-S′. It was significantly improved in the intra-group changes during these periods.

Assessment of RV function using conventional echocardiography is challenging due to the complex geometry of the RV and the predominantly longitudinal orientation of its myofibrils.^28^,^29^ Therefore, we used a novel technique, 2D-STE, which is a sensitive, quantitative measure of contractility, emerging as a potent measure of RV function that can determine RV systolic dysfunction.^30^,^31^ Strain can be decreased even in the setting of normal contractility if regional or global stress such as afterload is elevated. This is even more pronounced in the setting of RV circulation, which is especially sensitive to afterload elevation and may be useful for evaluating subtle changes compared with other conventional echocardiographic techniques.^13^

Peak RV longitudinal strain, which quantifies the maximal shortening in the RV free wall from apex to base, is likely to be a good estimator of RV function because 80% of the stroke volume is generated by longitudinal shortening of the RV free wall.^32^ In our study RV-free-S and RV-free-SR means were similar in the early period. Mean regional and mean RV freewall strain/strain rates observed at the one-month follow up were significantly increased compared to the pre-PCI period within each individual group.

According to conventional methods, sensitive changes in RV circulation can be detected earlier with 2D-STE.^33^ Since evaluation of the RV with conventional echocardiography after acute MI is not always possible, 2D-STE may be useful in this regard.

RV dysfunction is an independent predictor of adverse prognosis after acute MI. The involvement of the RV during inferior acute MI has been defined as a strong predictor of morbidity and in-hospital mortality.^7^,^8^ Previous studies described proximal RCA occlusion compromising flow to the major RV branches as the most common anatomical substrate for RV dysfunction.^34^-^36^ However our study confirmed that the location of the proximal RCA lesion was similar between the study groups.

In the study by Mehta *et al.*, post-PCI, TIMI 0–2 flow rate was reported as 14.7%.^37^ Brosh et al. reported TIMI 0–1 flow rates as 6.7%.^38^ In the study by Henriques *et al.*, however, no reflow was 11%.^39^ Although initial TIMI 0, TIMI 1–2 and TIMI 0–2 flow rates were 1.9, 6.2 and 8.1%, respectively in our study, similar results to these studies are available. There was no statistical difference between the groups in terms of TIMI flow rates.

In a recent study, ventricular tachycardia (VT) and fibrillation rates were higher in the TT group. Patients were monitored by event recorder monitors in the intensive care unit and service follow ups. Therefore, reperfusion arrhythmias such as accelerated idioventricular rhythm were excluded in the VT evaluation. The rates given in the tables were documented from the time of admission to hospital until discharge. However re-MI, re-hospitalisation and mortality rates were similar in both groups in hospital and at the one-month follow up.

Patients should be revascularised as early as possible in order to minimise potential complications following RV-STEMI. RV function may recover within days or weeks, especially after successful reperfusion.^40^-^43^ The findings of our study demonstrated similar improvement in RV dimensions and volumes among patients treated with PPCI, or PCI within three to 12 hours after TT. This results from the rapid improvement in RV systolic function once revascularisation is achieved. Both the PPCI and TT groups exhibited near-normal values for the parameters at the one-month follow up compared to at admission.

## Limitations of the study

The main limitation of this study is its relatively small sample size. Our study included the in-hospital and one month after RV-STEMI periods only, and may therefore have failed to capture differences in relevant parameters. For this study, a group had been planned to include patients undergoing PCI within 12 to 24 hours of TT; however, the number of patients revascularised at our centre during this time period was insufficient for statistical analysis.

Because the patients in the TT group had been referred from external centres, echocardiographic evaluation prior to thrombolytics was not possible. Therefore the RV systolic function determined in the TT group may have been overestimated.

RV strain was assessed by 2D speckle-tracking echocardiography software, which has been developed mainly for LV strain. However, investigators demonstrated that the reproducibility of RV strain was acceptable using a speckletracking programme for LV strain.

Moreover, RV septal and RV free wall were not separately evaluated during the RV strain analysis. However, previous studies suggested that RV septal strain showed no association with RV functional parameters. A possible explanation is that current speckle-tracking software cannot accurately separate LV septal from RV septal components, because the latter includes both RV and LV functional components.

In our study, we could not evaluate 2D-derived estimation of RVEF because of the heterogeneity of methods and the numerous anatomical assumptions. We did not have 3D software during the study period.

## Conclusions

Our study included inferior STEMI with RV involvement alone. RV function, which evaluated conventional and RV strain/strain rates, was similar between the two groups at admission and at the one-month follow up. In light of these results, we believe that revascularisation within three to 12 hours from TT may be as beneficial as PPCI for restoring RV systolic function. RV-STEMI diagnosis should be prompt and TT should be initiated in centres where PPCI cannot be performed. Patients should then immediately be referred to centres with coronary laboratories.
